# Mental Wellbeing of Family Members of Autistic Adults

**DOI:** 10.1007/s10803-017-3269-z

**Published:** 2017-08-31

**Authors:** Renske Herrema, Deborah Garland, Malcolm Osborne, Mark Freeston, Emma Honey, Jacqui Rodgers

**Affiliations:** 1 Clinical Psychology, Institute of Neuroscience, Faculty of Medical Sciences, Newcastle University, Ridley Building, Newcastle Upon Tyne, NE1 7RU UK; 2 National Autistic Society, Newcastle Upon Tyne, UK; 3 The Kayaks Support Group, Tyne & Wear, UK; 4 School of Psychology, Faculty of Medical Sciences, Newcastle University, Newcastle Upon Tyne, UK; 5 Northumberland, Tyne and Wear NHS Foundation Trust, Newcastle Upon Tyne, UK

**Keywords:** Adults, Autism, Family members, Mental health, Wellbeing

## Abstract

Family members are often the primary caregiver for autistic adults and this responsibility may impact on the carer’s wellbeing and quality of life. 109 family members of autistic adults completed an online survey assessing their wellbeing relating to their caring role for their autistic relative. Family members who were supporting an autistic relative with co-occurring mental health difficulties and who they reported as unprepared for the future, self-reported higher levels of worry, depression, anxiety and stress, and poorer quality of life. These findings emphasise the importance of support for family members of autistic adults, whether through external services to support their relative or individual mental health support for the carer.

## Introduction

As autistic individuals approach adulthood, external support often diminishes and family members frequently become the primary source of support (Happé and Charlton 2012; Howlin and Moss 2012). Research shows this increased responsibility can lead to stress among family members and within households (Benderix and Sivberg 2007; Rao and Beidel 2009; Smith et al. 2012), with family members, for example, reporting the need to restrict certain aspects of family life such as family days out and holidays (Hutton and Caron 2005; Montes and Halterman 2007). In addition, research has shown autistic adults are at increased risk of experiencing mental health problems, with anxiety difficulties reported as common amongst autistic adults and their family members (Davis et al. 2011; Van Bourgondien et al. 2014). Sterling et al. (2008) report in a sample of autistic 18–44 year olds that around 35% had anxiety, whilst Mazefsky et al. (2008) report 77% of autistic adults in their sample met criteria for an anxiety disorder. When mental health difficulties are present, this can be an additional barrier in addition to autism specific barriers towards independence for autistic adults (Smith and Philippen 2005), further increasing the need for support from others.

### Family Members/Caregivers of Autistic Adults

Hare et al. (2004) interviewed families of 26 autistic adults, and found parental emotional distress was prominent and directly associated with unmet need of the autistic family member. One of the unmet needs identified by family members was the capability to plan for their relatives’ future and the majority of participants expressed concern about the future for their autistic relative, due to the lack of service provision. Krauss et al. (2005) explored the positive and negative experiences of mothers of autistic adults. They directly compared those who lived in the family home versus those in residential care. Those whose relative lived with them in the family home reported experiencing daily stress, whereas for those whose relative was in residential care, family members reported worry and concern about their relative’s needs being met, as well as guilt that they were no longer caring for them. Bitsika and Sharpley (2004) report two-thirds of parents of autistic children in their sample were clinically depressed, further reinforcing the mental health needs of family members of autistic individuals.

Dillenburger and McKerr (2011) explored long-term care and support available for parents and caregivers of adults with intellectual or developmental disabilities and found a gap in services and lack of future planning, which can cause increased stress for caregivers. Uncertainty about the future is a concern for many family members of autistic adults. Farley and McMahon (2014) highlight that caregivers can often be unprepared in terms of accessing external services to support their relative before it is unavoidable, due to them no longer being able to provide care.

When parents are no longer able to care for their autistic relatives, it is often siblings or other family members who become the primary caregiver. Arnold et al. (2012) explored the needs of siblings of individuals with developmental disabilities, particularly those taking over as their primary caregiver in the future. Siblings described the need for support services to address their own concerns, as they often find themselves alone or isolated due to time spent caregiving. This is further supported by Benderix and Sivberg (2007), who found that siblings of autistic individuals with an intellectual disability experienced stressful life conditions and limited their own social lives, which they attributed to their caring role. The current literature therefore substantiates the stress and strain and the prevalence of mental health difficulties amongst those caring for an autistic relative. The predictors and drivers of these mental health difficulties are less clear.

Difficulty tolerating uncertainty about the future has been identified as a major contributor to the development and maintenance of anxiety disorders (Carleton 2012). Evidence is increasing that intolerance of uncertainty (IU) is a major driver for increased anxiety among autistic people (Boulter et al. 2014; South and Rodgers 2017; Maisel et al. 2016). IU is considered to be a ‘broad dispositional risk factor for the development and maintenance of clinically significant anxiety’ (Carleton 2012). It involves the ‘tendency to react negatively on an emotional, cognitive and behavioural level to uncertain situations and events’ (Buhr and Dugas 2009). Individuals who are intolerant of uncertainty find uncertain situations stressful and upsetting; have a tendency to interpret all ambiguous information as threatening and find it difficult to function in the face of uncertainty (Buhr and Dugas 2002, 2009; Laugesen et al. 2003). Indeed, uncertainty itself is perceived as threatening by people high in IU (Freeston et al. 1994; Carleton 2012). Given that autistic adults and their family members face uncertainty with regard to the future, we examined the relationships between intolerance of uncertainty and mental health problems among family members and caregivers of autistic adults.

The aim of the current research was therefore to investigate the mental wellbeing of family members providing support to autistic adults and to identify specific factors that may predict poorer wellbeing outcomes for family members. It is predicted that some of the challenges caring for an autistic adult such as the presence of intellectual disabilities, challenging behaviour and additional mental health problems as well as uncertainties about the future will contribute to poorer mental health outcomes for family members, namely worry, depression, anxiety and stress. Further, family members who have higher levels of intolerance of uncertainty will have higher levels of mental health symptoms themselves.

## Methodology

### Design and Analysis

The design was a single group online survey based design. An online survey was selected in order to reach participants across the United Kingdom. The survey results were analysed using qualitative and quantitative analysis. Validated measures of wellbeing were used in order to appropriately collect accurate scores regarding family member’s mental health status.

### Participants

**Fig. 1:**
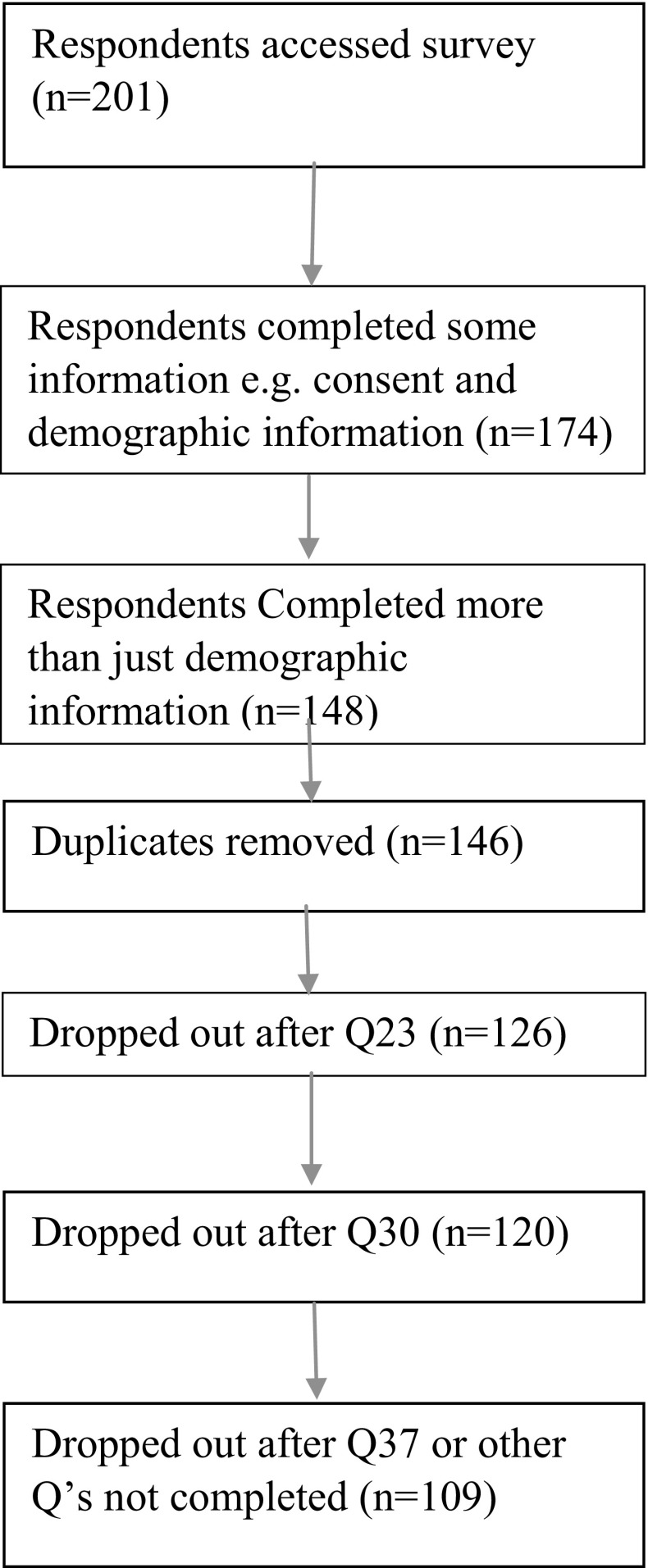
CONSORT diagram showing respondents completion for online survey data analysis

109 family members of autistic adults completed an online survey designed specifically for the current study. Participants were largely recruited through the Adult Autism Spectrum Cohort (AASC-UK), a database hosted at Newcastle University. AASC-UK provides opportunities for autistic adults and their relatives to participate in research (http://research.ncl.ac.uk/adultautismspectrum/). Participants were also recruited through the following associations; Research Autism, Scottish Autism, the National Autistic Society and The North East Autism Society. The CONSORT diagram shows respondent completion rates, the initial drop-out was the largest, whereby participants clicked on the survey link but did not complete any information or only their own demographic information (n = 53). This drop-out rate may have been due to respondents accessing the survey and realising they were not eligible to take part (e.g. a parent of an autistic child rather than an autistic adult). (Insert Fig. 1 here).

### Procedure

There were 45 groups of questions in the survey which took around 30 minutes to complete. The survey was designed to retrieve information about the family members and caregivers supporting an autistic adult, their relationship to the adult they were supporting, as well as additional information about the adult themselves. Participants then completed several validated questionnaires; Intolerance of Uncertainty Scale—Parent (IUS-P), Intolerance of Uncertainty Scale (IUS-12), Penn State Worry Questionnaire (PSWQ), the Depression Anxiety and Stress Scale (DASS-21) and a quality of life measure. The survey was presented on Qualtrics™. The design, content and formatting were adapted based on feedback from members of the research team, including an adult with Asperger’s syndrome and a parent of an autistic adult. Potential participants were directed to the survey through the AASC-UK cohort, charities, service providers, websites, e-mails and social media advertising the web link and information about the study.

### Measures

#### Intolerance of Uncertainty Scale (IUS-12)

The IUS-12 is a short 12-item questionnaire which assesses anticipatory and inhibitory components of Intolerance of Uncertainty (Carleton et al. 2007). The 12 items are rated on a 5 point Likert scale ranging from 1 (not at all characteristic of me) to 5 (entirely characteristic of me). The sum of the total scores as indicated by the participants’ responses reflects the degree of their intolerance to uncertain events/situations. Buhr and Dugas (2002) report the IUS-12 to have excellent internal consistency and good test retest reliability.

#### Intolerance of Uncertainty Scale—Parent (IUS-P)

The IUS-P is a short 12-item questionnaire which is adapted from the IUS-12 and designed for parents or caregivers to answer on behalf of the individual they are supporting. The items are the same as that of the IUS-12 but allow the respondent to report on the level of intolerance to uncertainty of the person they are caring for. Here respondents reported on the autistic adult they were supporting.

#### Penn State Worry Questionnaire (PSWQ)

PSWQ is a 16-item questionnaire which measures the trait of worry (Meyer et al. 1990). The 16 items are rated on a 5 point Likert scale ranging from 1 (not at all typical of me) to 5 (very typical of me). Research shows that this scale can discriminate those with generalized anxiety disorder compared with any other anxiety disorder. This scale has been shown to have high internal consistency and good test retest reliability (Meyer et al. 1990).

#### Depression, Anxiety and Stress Scale (DASS-21)

The DASS-21 (Lovibond and Lovibond 1995) is a short-form 21 item self-report questionnaire which measures the severity of a range of symptoms common to depression, anxiety and stress. In completing the DASS-21, the individual is required to indicate the presence of a symptom over the previous week. Each item is scored from 0 (did not apply to me at all over the last week) to 3 (applied to me very much or most of the time over the past week). The DASS-21 assesses the severity of the core symptoms of depression, anxiety and stress. Crawford and Henry (2003) report the reliability of the DASS-21 to be excellent.

#### Quality of Life Measure

The brief quality of life measure was derived by collating themes from 11 different standardised quality of life measures, which are frequently used in the typically developing population. This resulted in seven main themes relating to quality of life; physical health, mental health, relationships with others, finances, residence, access to support, positivity about the future, sense of fulfilment and overall satisfaction with life. Therefore, this quality of life measure had 7 items which participants rated on a 5 point Likert scale from 1 (very poor) to 5 (very good).

#### Ethical Approval

A favourable ethical opinion for the study was provided by the Ethics Committee of the Faculty of Medical Sciences, Newcastle University, UK. Participants were given detailed information about what the survey would involve, contact information for local support, and how to contact the Primary Investigator (JR) of the research. Participants were requested to indicate their consent on the first webpage prior to being able to access the actual survey. Participants were made aware of the confidentiality procedure and that their contributions were completely anonymous. On exiting the survey, participants were presented with debriefing information and contact details for relevant support services.

## Results

The results outlined show the respondent demographic information and demographic information about their autistic family member whom they support. The respondents’ mental wellbeing scores are then outlined and predictors of these scores are examined using multiple hierarchical regression.

### Respondent Demographic Information

Table 1 shows demographic information for family members of autistic adults. The mean age of respondents was 54 ± 9 (range 25–71 years).

**Table 1:** Respondent demographics (N = 109)

Category	Categories	Frequency	%
Gender	Male	14	13
	Female	95	87
Marital status	Single, never married	11	10
	Married or domestic partnership	82	75
	Widowed/divorced/separated	16	15
Education	Did not complete high school	7	6
	GCSE’s or equivalent	18	17
	A levels/college qualifications	28	26
	Bachelor’s degree or above	53	49
	Not sure	3	2
Employment	Employed	51	47
	Unemployed	2	2.5
	Retired	20	18
	Student	1	1
	Full time home-maker/carer	28	25
	Unable to work/disabled	5	4
	Voluntary work	2	2.5
Relationship to individual on the autism spectrum	Mother	79	72
	Father	11	10
	Sibling	9	8
	Carer	2	1
	Spouse	4	4.5
	Other, please specify	4	4.5

### Demographic Information About the Autistic Family Member

Table 2 outlines the demographic information about the autistic adult (known as “X”) for whom the respondents were providing care. The mean age of the autistic relative was 27 ± 9 (range 18–67 years). Respondents stated that the mean age that their autistic relative received their diagnosis of ASD was 16 ± 13 (range 1 month–60 years old). Seventy-seven percent (n = 84) of autistic individuals were also reported to have a mental health problem (co-morbid anxiety and/or depression or another mental health difficulty), whereas 16% (n = 18) had another disorder/difficulty with no mental health difficulty, and only 6% (n = 7) were reported to have no additional difficulties/disorders. Seventy-two percent of the autistic adults were reported by their relative to have co-morbid anxiety and 39% were reported to have co-morbid depression.

**Table 2:** Demographic information about autistic family member (N = 109)

Question	Categories	Frequency	%
Gender	Male	78	72
	Female	31	28
Diagnosis	Autism	24	22
	Autism spectrum disorder (ASD)	31	28
	Asperger’s syndrome	42	39
	Pervasive developmental disorder—not	1	1
	Otherwise specified (PDD-NOS)		
	Other, please specify	11	10
Co-morbid difficulties (please tick all that apply)	Intellectual/learning disability	38	35
	Attention deficit hyperactivity disorder (ADHD)	17	16
	Anxiety	79	72
	Depression	43	39
	Epilepsy	11	10
	Sleep disorders	24	22
	Challenging behaviours	38	35
	Other mental health disorder	16	15
	Other, please specify	27	25
	No additional difficulties	7	6
Co-morbid difficulties (collapsed categories)	Mental health difficulty (anxiety/depression/stated eating disorder etc.)	84	77
	Other difficulty with NO mental health difficulty (e.g. LD, ADHD, epilepsy, sleep, disorder, challenging behaviour)	18	16
	No difficulty	7	6
Marital status	Single, never married	98	90
	Married or domestic partnership	7	6
	Divorced	1	1
	Separated	3	3
Living arrangements	In the family home	66	61
	In supported accommodation	9	8
	In a residential care home	6	5
	Living independently	18	17
	Other, please specify	10	9
Employment status (please tick all that apply)	Unemployed	44	40
	Studying at college/university	27	25
	Working full-time/part-time	19	17
	Doing volunteer work	12	11
	Attending day care centre	6	5
	Other, please specify	30	28
Main source of support	Respondent	74	68
	Other family member	17	16
	Carer	1	1.5
	Support worker	8	7
	Personal assistant	1	1.5
	External service	7	6

### Mental Health of Family Members Providing Support for Adults on the Autism Spectrum

Mean scores (SD and range) are presented for each of the questionnaires in Table 3. 80% of respondents rate their autistic relative to be within the clinical range for levels of intolerance of uncertainty [indicative cut-off of 35 (Carleton 2012b)]. The respondents rated their own levels of intolerance of uncertainty, with a mean of 29.01 ± 11.08 for the IUS, with 29% of respondents within the clinical range. The means scores for the DASS-21 show mild levels of depression with a mean of 12.63 ± 5.36, with 60% of respondents reporting mild or above depression levels, moderate levels of anxiety with a mean of 11.03 ± 4.41, with 73% reporting mild or above anxiety levels and normal levels of stress with a mean of 14.80 ± 5.04, with 47% reporting mild or above stress levels. The PSWQ mean score of 51.09 ± 13.89 shows respondents are on average reporting moderate levels of worry and 75% of respondents’ scores were in the moderate to severe range.

**Table 3:** Mean scores from mental wellbeing measures

Questionnaire	N	Min	Max	Mean	SD	Cut score	% above cutoff
Autistic person							
Intolerance of uncertainty scale—P (rated by carer)	104	12	60	44.81	10.74	35	80% indicative
Family member							
Intolerance of uncertainty scale (IUS-12)	108	13	60	29.01	11.08	35	29% indicative
Depression, anxiety and stress scale (DASS-21)							Mild or above
Depression	108	7	27	12.63	5.36	10	60%
Anxiety	109	7	24	11.03	4.41	8	73%
Stress	108	7	28	14.80	5.04	15	47%
Penn state worry questionnaire	109	25	79	51.09	13.89	40	75% (Moderate-severe)
Quality of life measure	108	18	45	31.06	6.74	–	–

### Quality of Life Measure

Cronbach’s alpha of 0.88 indicates a high level of internal consistency for this scale within this sample. Independent sample *t* tests showed that those who cared for an autistic adult with a co-morbid mental health difficulty (n = 84) reported a significantly poorer quality of life (M = 30.02 ± 6.61) than those who cared for someone without a mental health difficulty (n = 25, M = 34.48 ± 6.11), t(106) = 3.01, p = .003).

### Associations

Correlations between the mental wellbeing scores were calculated and are reported in Table 4. There were significant, moderate, positive correlations between the mental health measures and negative correlations with Quality of Life. The IUS-P scores for the autistic adult (rated by their family member) were lower and in the case of depression, not significant. Tables 5, 6, 7 and 8.

**Table 4:** Associations between mental health measures

Correlations						
	IUS	Depression	Anxiety	Stress	PSWQ	QoL
Autistic person						
IUS-P	0.242	0.165	0.272	0.353	0.266	−0.276
Family member						
IUS	–	0.534	0.593	0.607	0.647	−0.413
DASS-depression		–	0.671	0.694	0.517	−0.659
DASS-anxiety			–	0.673	0.596	−0.466
DASS-stress				–	0.699	−0.518
PSWQ					–	−0.461

N = 103–109; p < .05

**Table 5:** Predictors of family member worry (PSWQ)

Step	Variable	df. num	df denom.	ΔR^2^	F	Sig. F	*β*	t	Sig. t
1		3	99	0.029	0.99	0.399			
	Age						−0.022	−0.22	0.827
	ID						−0.122	−1.19	0.237
	Chall. Behav						0.147	1.45	0.151
2		**2**	**97**	**0.070**	**3.77**	**0.026**			
	Mental health						0.168	1.74	0.085
	Preparedness						−**0.207**	−**2.12**	**0.037**
3	IUS-P	**1**	**96**	**0.038**	**4.27**	**0.041**	**0.215**	**2.07**	**0.041**
4	Family member IUS	**1**	**95**	**0.342**	**62.44**	<**0.001**	**0.606**	**7.90**	**0.000**

Bold values represent the significance levels

**Table 6:** Predictors of family member depression (DASS-depression)

Step	Variable	df. num	df denom.	ΔR^2^	F	Sig. F	*β*	t	Sig. t
1		3	99	0.025	0.863	0.463			
	Age						−0.061	−0.609	0.544
	ID						−0.098	−0.958	0.340
	Chall.. Behav						0.133	1.302	0.196
2		**2**	**97**	**0.070**	**3.748**	**0.027**			
	Mental health						**0.246**	**2.537**	**0.013**
	Preparedness						−0.100	−1.017	0.312
3	IUS-P	1	96	0.005	0.519	0.473	0.076	0.720	0.473
4	Family member IUS	**1**	**95**	**0.251**	**36.838**	<**0.001**	**0.519**	**6.069**	**0.000**

Bold values represent the significance levels

**Table 7:** Predictors of family members anxiety (DASS-anxiety)

Step	Variable	df. num	df denom.	ΔR^2^	F	Sig. F	*β*	t	Sig. t
1		3	99	0.027	0.904	0.442			
	Age						0.078	0.755	0.440
	ID						−0.081	−0.786	0.434
	Chall. Behav.						0.138	1.354	0.179
2		**2**	**97**	**0.129**	**7.434**	**0.001**			
	Mental health						**0.284**	**3.033**	**0.003**
	Preparedness						−**0.224**	−**2.367**	**0.020**
3		1	96	0.026	3.018	0.086			
	IUS-P						0.176	1.737	0.086
4		**1**	**95**	**0.280**	**49.549**	**0.000**			
	Family member IUS						**0.549**	**7.039**	**0.000**

Bold values represent the significance levels

**Table 8:** Predictors of  family members stress (DASS-stress)

Step	Variable	df. num	df denom.	ΔR^2^	F	Sig. F	*β*	t	Sig. t
1		3	99	0.034	1.166	0.327			
	Age						−0.071	−0.708	0.480
	ID						−0.096	−0.937	0.351
	Chall.. Behav						0.164	1.620	0.108
2		**2**	**97**	**0.112**	**6.392**	**0.002**			
	Mental health						**0.203**	**2.153**	**0.034**
	Preparedness						−**0.271**	−**2.845**	**0.005**
3	IUS-P	**1**	**96**	**0.068**	**8.295**	**0.005**	**0.285**	**2.880**	**0.005**
4	Family member IUS	**1**	**95**	**0.261**	**47.391**	**0.000**	**0.530**	**6.884**	**0.000**

### Multiple Hierarchical Regression

Secondly, in order to examine predictors of carer’s mental health, a series of hierarchical multiple regression analyses were undertaken. Predictors included age of the autistic adult for whom support was provided, whether or not the autistic adult had an intellectual disability, whether or not the autistic adult exhibited challenging behaviour or had a co-occurring mental health difficulty, how prepared they were for the future (as rated by the family member) and the autistic adult’s level of intolerance of uncertainty. Finally the family member’s intolerance of uncertainty was also entered. Criterion variables were PSWQ total score and Depression, Anxiety and Stress sub-scale scores from DASS-21.

### Predictors of Family Member Worry

At step 1, the autistic person’s age, presence or not of an intellectual disability and presence of challenging behaviour were entered but did not make a significant contribution to the prediction of family member worry. The presence of mental health problems and the autistic adult’s preparedness for the future were entered next and accounted for 7% of the variance, but only preparedness for the future of the autistic adult made a significant individual contribution; less preparedness of the autistic adult was associated with higher carer worry. Next, the IUS-P score of the autistic person was entered at step three and made a significant contribution to family member worry, accounting for 3.8% of the variance. Finally the family members’ IUS score was entered and made a significant contribution accounting for 34.2% of the variance. The overall model accounted for 48.0% of the variance, (R^2^ adj = 44.1%), F (7, 95) = 12.511, p < .001).

### Predictors of Family Member Depression

The presence of a mental health disorder in the autistic adult they were supporting predicted a significant increase in depression score within Step 2 accounting for 7.0% of the variance but IUS-P at step 3 did not. The carer’s IUS score in Step 4 to the model was the main significant contribution, accounting for 25.1% of the variance, meaning family members’ higher levels of intolerance of uncertainty predicted higher levels of depression. The overall model accounted for 35.2% of the variance (R^2^ adj = 30.4%), F (7, 95) = 7.361, p < .001).

### Predictors of Family Member Anxiety

At Step 2, the presence of a mental health disorder in the autistic adult they were supporting and their preparedness for the future were significant predictors of family member anxiety, accounting for 12.9% of the variance. The addition of family member IUS made a significant contribution to family member anxiety, accounting for 28.0% of the variance. The overall model accounted for 46.2% of the variance (R^2^ adj = 42.3%), F (7, 95) = 11.665, p < .001).

### Predictors of Family Member Stress

At Step 2 co-morbid mental health difficulties of the autistic adult they were supporting and their preparedness for the future were significant predictors of family member stress and the model accounted for 11.2% of the variance. The levels of intolerance of uncertainty (IUS-P) of the autistic family member also made a significant contribution, accounting for 6.8% of the variance. Lastly, the addition of family member IUS was a significant predictor of carer stress, accounting for 26.1% of the variance. The overall model accounted for 47.6% of the variance (R^2^ adj = 43.7%), F (7, 95) = 12.324, p < .001).

## Discussion

Our aims were to explore the predictors of mental wellbeing amongst family members and caregivers supporting an autistic adult. The results from this research show that symptoms indicative of possible mental health difficulties are indeed prevalent among family members caring for autistic adults, with two-thirds indicating scores indicative of mild or moderate depression, three quarters reporting anxiety and worry and one-half self-reporting clinical levels of stress. These findings support previous research showing mental health difficulties are often prominent in family members caring for autistic individuals (Davis et al. 2011; Van Bourgondien et al. 2014). The predictors of lower mental wellbeing for family members were the presence of mental health difficulty, level of IU and preparedness for the future for the autistic adult they were supporting, as well as family members’ own intolerance of uncertainty. According to our findings, the presence of intellectual disability or challenging behaviours amongst autistic adults did not significantly contribute to family member mental wellbeing in this sample, whereas the mental health of the individual receiving support is a significant predictor of family member wellbeing. It may be of course, that these relationships are bi-directional and cyclical in nature suggesting that mental health support needs to be in place for both family members and autistic individuals to break this cycle. The consequences of this unmet need may be profound. Research suggests that suicide rates are much higher amongst autistic adults than the general population (Cassidy et al. 2014; Farley and McMahon 2014) further emphasizing the importance of timely mental health support for these individuals and the extreme costs that mental health difficulties can have for autistic individuals and their family members.

We found that lack of preparedness of the autistic individual for the future significantly predicted higher levels of worry, anxiety and stress for the caregiver. It will be important in future research to explore in more detail barriers to preparation for the future. Likewise, future research should investigate what provision for future planning could be made available for autistic adults and their family members, to enable them to feel more prepared for the future. If services and support enabled autistic adults to increase their independence in terms of residence, employment and finances etc., family members and the adults themselves may feel more at ease in terms of looking toward the future.

These results are consistent with Farley and McMahon (2014) who highlighted preparedness for the future as key in wellbeing for autistic adults and their families. They also support the National Autistic Society’s “Getting on? Growing older with autism” (2013), which outlines the importance of preparing for the future and providing information, support and services for autistic adults. Interestingly, the degree to which family members had made plans for their relatives’ future was not a significant predictor of poorer outcomes for the family members. This further highlights that in this sample it is the readiness or preparedness of the autistic individual they are supporting that predicts higher levels of worry, anxiety and stress, and thus, enabling autistic adults to feel prepared for their own future is of top priority in order to address this for family members’ wellbeing.

We found that level of IU amongst the autistic adults was also a significant predictor of family member wellbeing, crucially of their worry and stress. As discussed previously, intolerance of uncertainty has been found to be an important mechanism in anxiety and autism (Boulter et al. 2014; South and Rodgers 2017). 72% of autistic adults in this population were reported to have co-morbid anxiety difficulties and 80% were reported by their family member to show levels of intolerance of uncertainty typically found among those with anxiety disorders, further supporting the emerging literature about the prevalence of IU and co-morbid anxiety difficulties among autistic adults. Interventions targeting IU for autistic adults may therefore be beneficial to the adults themselves and to those supporting them.

This was the first study to our knowledge to explore IU amongst carers of autistic adults. We found that self-reported family member IU was a significant and strong predictor, accounting for 25–34% of the variability (when entered after the autistic person’s characteristics) in worry, depression, anxiety and stress. This supports evidence from previous research (Buhr and Dugas 2012, 2006, 2009; Dugas et al. 1997, 2005; Freeston et al. 1994) which shows that IU plays an integral role in the development and maintenance of mental health problems in the general population and should therefore be addressed more directly. Hare et al. (2004) showed that caring for an autistic individual could have a negative impact on wellbeing on the caregiver due to the stressors and strain of this responsibility. As IU interacts with a range of factors associated with the caring role, it may be crucial to address family member IU in future interventions.

The findings have clear clinical implications. Autistic adults with mental health needs require access to appropriately informed mental health services. They will also need services to enable greater preparedness for the future. Together, these may serve to reduce their own mental health difficulties and also reduce the impact on family members supporting autistic adults. This in turn may have downstream benefits for the whole family.

These findings in relation to family members and previous findings about autistic people indicate that IU makes an important contribution to mental health difficulties for both autistic individuals and their caregivers/family members. IU in the lives of autistic adults and their family members can be addressed in two ways. Firstly, service provision can serve to decrease some of uncertainty regarding the future by providing clear routes to information and pathways and models of care, thus enabling better preparation and planning for the future.

Of course, even with better provision there will still inevitably be some uncertainty in life and so secondly, interventions targeting intolerance to uncertainty may therefore be appropriate. Rodgers et al. (2016) report the development of a parent mediated group intervention specifically targeting intolerance of uncertainty for autistic children, Coping with Uncertainty in Everyday Situations (CUES©). Successful implementation of this intervention with parents of autistic children suggest that targeting IU directly has a beneficial impact on both child and parent IU and anxiety. Preliminary results have shown that adapting this programme for use with autistic adults (CUES-A©) is also feasible (*paper in prep*.). By directly targeting IU with autistic adults, we may see improvements in mental health, and perhaps also in the mental health of the family member supporting them, especially if family members are involved at specific points in treatment.

Limitations of this research include a relatively narrow population sample, as participants were recruited through their involvement in organisations addressing the concerns of autistic people and their families and their interest in research. For example, they may be particularly attuned to mental health issues, although other studies support the presence of mental health difficulties among autistic people and their caregivers alike (Bradley et al. 2004; Hare et al. 2004). In addition, the majority of respondents were mothers of the autistic adults and thus it is mostly their views and mental wellbeing which are represented here. It would be beneficial for future research to identify a more representative sample of all family members. Our data may not represent the full range and diversity of difficulties which can be prevalent across the autistic spectrum. Further research should work towards identification of the specific support necessary to promote wellbeing for caregivers and independence and wellbeing for autistic adults. This would enable support to be more specific and tailored to address unmet need in adulthood for autistic adults and their family members. Future research could also explore any further variables which may contribute to poorer mental health outcomes in family members and autistic adults. For example, this project did not explore the impact of environmental factors such as socio-economic status and ethnicity of participants. By further operationalising predictors of poorer mental health among family members of autistic adults, support can be adapted to be more effective and efficient for this population.

Our findings demonstrate that family members who support autistic adults with co-morbid mental health difficulties may be at increased risk of mental health difficulties themselves and that “preparedness for the future” for the autistic adult contributes towards caregiver worry, anxiety and stress. Furthermore intolerance of uncertainty for both the autistic adult and their caregiver makes an additional contribution to caregiver mental health difficulties. Service provision which aims to reduce uncertainty wherever possible, alongside interventions to increase tolerance to uncertainty may best serve the mental health needs of these families and their autistic relatives.

## References

[CR2] Arnold CK, Heller T, Kramer J (2012). Support needs of siblings of people with developmental disabilities. Intellectual and Developmental Disabilities.

[CR4] Benderix Y, Sivberg B (2007). Siblings’ experiences of having a brother or sister with autism and mental retardation: a case study of 14 siblings from five families. Journal of Pediatric Nursing.

[CR5] Bitsika V, Sharpley CF (2004). Stress, anxiety and depression among parents of children with autism spectrum disorder. Australian journal of guidance and counselling.

[CR9] Boulter C, Freeston M, South M, Rodgers J (2014). Intolerance of uncertainty as a framework for understanding anxiety in children and adolescents with autism spectrum disorders. Journal of Autism and Developmental Disorders.

[CR10] Bradley EA, Summers JA, Wood HL, Bryson SE (2004). Comparing rates of psychiatric and behavior disorders in adolescents and young adults with severe intellectual disability with and without autism. Journal of autism and developmental disorders.

[CR12] Buhr K, Dugas MJ (2002). The intolerance of uncertainty scale: Psychometric properties of the English version. Behaviour Research and Therapy.

[CR13] Buhr K, Dugas MJ (2006). Investigating the construct validity of intolerance of uncertainty and its unique relationship with worry. Journal of Anxiety Disorders.

[CR14] Buhr K, Dugas MJ (2009). The role of fear of anxiety and intolerance of uncertainty in worry: An experimental manipulation. Behaviour Research and Therapy.

[CR15] Buhr K, Dugas MJ (2012). Fear of emotions, experiential avoidance, and intolerance of uncertainty in worry and generalized anxiety disorder. International Journal of Cognitive Therapy.

[CR16] Carleton RN (2012). The intolerance of uncertainty construct in the context of anxiety disorders: Theoretical and practical perspectives. Expert Review of Neurotherapeutics.

[CR19] Carleton RN, Sharpe D, Asmundson GJ (2007). Anxiety sensitivity and intolerance of uncertainty: Requisites of the fundamental fears?. Behaviour Research and Therapy.

[CR20] Cassidy S, Bradley P, Robinson J, Allison C, McHugh M, Baron-Cohen S (2014). Suicidal ideation and suicide plans or attempts in adults with Asperger’s syndrome attending a specialist diagnostic clinic: a clinical cohort study. The Lancet Psychiatry.

[CR21] Crawford JR, Henry JD (2003). The depression anxiety stress scales (DASS): Normative data and latent structure in a large non-clinical sample. British Journal of Clinical Psychology.

[CR22] Davis TE, Hess JA, Moree BN, Fodstad JC, Dempsey T, Jenkins WS, Matson JL (2011). Anxiety symptoms across the lifespan in people diagnosed with autistic disorder. Research in Autism Spectrum Disorders.

[CR23] Dillenburger K, McKerr L (2011). ‘How long are we able to go on?’Issues faced by older family caregivers of adults with disabilities. British Journal of Learning Disabilities.

[CR24] Dugas MJ, Freeston MH, Ladouceur R (1997). Intolerance of uncertainty and problem orientation in worry. Cognitive Therapy and Research.

[CR26] Dugas MJ, Marchand A, Ladouceur R (2005). Further validation of a cognitive-behavioral model of generalized anxiety disorder: Diagnostic and symptom specificity. Journal of Anxiety Disorders.

[CR29] Farley M, McMahon B (2014). Range of outcomes and challenges in middle and later life. Adolescents and Adults with Autism Spectrum Disorders.

[CR30] Freeston MH, Rhéaume J, Letarte H, Dugas MJ, Ladouceur R (1994). Why do people worry?. Personality and Individual Differences.

[CR36] Happé F, Charlton R (2012). Mini review: Aging in autism spectrum disorders.

[CR37] Hare DJ, Pratt C, Burton M, Bromley J, Emerson E (2004). The health and social care needs of family carers supporting adults with autistic spectrum disorders. Autism: The International Journal of Research and Practice.

[CR42] Howlin P, Moss P (2012). Adults with autism spectrum disorders. Canadian Journal of Psychiatry. Revue Canadienne de Psychiatrie.

[CR43] Hutton AM, Caron SL (2005). Experiences of families with children with autism in rural New England. Focus on Autism and Other Developmental Disabilities.

[CR44] Kenny L, Hattersley C, Molins B, Buckley C, Povey C, Pellicano E (2016). Which terms should be used to describe autism? Perspectives from the UK autism community. Autism: The International Journal of Research And Practice.

[CR45] Krauss MW, Seltzer MM, Jacobson HT (2005). Adults with autism living at home or in non-family settings: Positive and negative aspects of residential status. Journal of Intellectual Disability Research.

[CR46] Laugesen N, Dugas MJ, Bukowski WM (2003). Understanding adolescent worry: The application of a cognitive model. Journal of Abnormal Child Psychology.

[CR47] Lovibond SH, Lovibond PF (1995). Manual for the Depression Anxiety Stress Scales.

[CR48] Maisel ME, Stephenson KG, South M, Rodgers J, Freeston MH, Gaigg SB (2016). Modeling the cognitive mechanisms linking autism symptoms and anxiety in adults. Journal of Abnormal Psychology.

[CR49] Mazefsky CA, Folstein SE, Lainhart JE (2008). Overrepresentation of mood and anxiety disorders in adults with autism and their first-degree relatives: What does it mean?. Autism Research.

[CR50] Meyer TJ, Miller ML, Metzger RL, Borkovec TD (1990). Development and validation of the penn state worry questionnaire. Behaviour Research and Therapy.

[CR51] Montes G, Halterman JS (2007). Psychological functioning and coping among mothers of children with autism: A population-based study. Pediatrics.

[CR52] National Autistic Society (2013) Getting on? Growing older with autism. http://network.autism.org.uk/sites/default/files/ckfinder/files/getting_on_-_policy_report.pdf. Accessed Sept 8 2016.

[CR56] Rao PA, Beidel DC (2009). The impact of children with high-functioning autism on parental stress, sibling adjustment, and family functioning. Behavior Modification.

[CR58] Rodgers J, Hodgson A, Shields K, Wright C, Honey E, Freeston M (2016). Towards a treatment for intolerance of uncertainty in young people with autism spectrum disorder: Development of the Coping With Uncertainty in Everyday Situations (CUES©) Programme. Journal of Autism and Developmental Disorders.

[CR62] Smith LE, Greenberg JS, Mailick MR (2012). Adults with autism: Outcomes, family effects, and the multi-family group psychoeducation model. Current Psychiatry Reports.

[CR63] Smith MD, Philippen LR, Zager D (2005). Community integration and supported employment. Autism spectrum disorders: Identification, education, and treatment.

[CR65] South M, Rodgers J (2017). Sensory, emotional and cognitive contributions to anxiety in autism spectrum disorders. Frontiers in Human Neuroscience.

[CR66] Sterling L, Dawson G, Estes A, Greenson J (2008). Characteristics associated with presence of depressive symptoms in adults with autism spectrum disorder. Journal of Autism and Developmental Disorders.

[CR67] Van Bourgondien ME, Dawkins T, Marcus L (2014). Families of adults with autism spectrum disorders. Adolescents and adults with autism spectrum disorders.

